# The first wave of COVID-19 and mental distress of physician residents in Brazil: a comparison between two cohorts

**DOI:** 10.1186/s12960-022-00790-5

**Published:** 2023-02-14

**Authors:** Mário Luciano de Mélo Silva Júnior, Arthur Violante Sapia, Jonas Marques Cavalcanti Neto, Nathallya Maria Gomes Barbosa, Victória Beatriz Costa Neiva, Euler Nicolau Sauaia Filho

**Affiliations:** 1 Division of Neuropsychiatry, Medical Science Center, Universidade Federal de Pernambuco, 1235 Moraes Rego Av, Cidade Universitária, Recife, 50670-901 Brazil; 2 Medical School, Uninassau, Recife, Brazil; 3 Neurology Unit, Hospital da Restauração, Recife, Brazil; 4 Instituto do Câncer do Estado de São Paulo, São Paulo, Brazil; 5 Medical School, Universidade Dom Bosco, São Luis, Brazil

**Keywords:** Depression, Anxiety, Burnout, Residency training, Workload

## Abstract

**Introduction:**

The reorganization of healthcare systems to face the COVID-19 pandemic has led to concerns regarding psychological distress of healthcare workers, and training requirements of physician residents.

**Objective:**

To assess the influence of COVID-19 pandemic on depression, anxiety, burnout and training schedules of residents.

**Methods:**

Two independent cross-sectional studies (the first in November 2019 [control], the second in June 2020, during the first wave of COVID-19 pandemic) enrolling physician residents from Brazil, using online surveys. In each of them, we collected demographic and training program data, and assessed depression, anxiety and burnout through PHQ-2, GAD-2 and MBI (2-item version) scales, respectively. We controlled confounding variables with logistic regression analysis.

**Results:**

The COVID-19 cohort (*n* = 524) presented a briefer workload and had at least 1 day off per week more frequently, in relation to the control cohort (*n* = 1 419). The majority of residents (464/524, 89.5%) had a reduction in their duty hours, and believed they would need an extra training period after the end of the pandemic (399/524, 76.2%). The frequency of depression increased (46.0% vs. 58.8%, aOR = 1.64, 95% CI = 1.32–2.05), anxiety did not change (56.5% vs. 56.5%, aOR = 1.24, 95% CI = 0.99–1.55) and burnout decreased (37.0% vs. 26.1%, aOR = 0.77, 95% CI = 0.60–0.99). Sensitivity analysis did not change these results.

**Conclusion:**

Mental distress is frequent among residents and associated with both training program and social environments. The consequences of the COVID-19 pandemic on training requirements should be specifically addressed by supervisors and policymakers, in a case-by-case basis. Psychological support must be provided to healthcare workers.

## Introduction

The COVID-19 pandemic has impacted the psychological health (stress, depression, anxiety, sleep disturbances and post-traumatic disorder) of health professionals [1, 2]. The high rates of mental distress in this population are related—but not limited—to intense workloads, ethical dilemmas, fear of infecting family members and therapeutic limitations found in the public health systems, especially in low- and middle-income countries [3, 4].

In Brazil, the plateau of the COVID-19 first wave occurred between June and August 2020, with an average of one thousand deaths per day [5]. At that time, the main worries were about insufficient human resources and consumables, such as personal protective equipment (PPE) and materials for taking care of COVID-19 patients, restrictive measures, social distancing, and its economic and social implications.

To face this public health urgency, many professionals were redeployed from their usual routines to COVID-19 frontline care, including the residents. In general, physician residents are young, inexperienced, and should perform their tasks under the supervision of qualified staff. Classically, residents have high rates of mental distress [6], which is related to the workload and the number of competencies and skills they need to attain in this training period. However, due to the changes and re-organizations made (mostly in secondary and tertiary centers, where residency programs take place) in order to handle COVID-19 demands, training schedules were negatively impacted [7], in tandem with increasing responsibilities of residents [8]. The consequences of those changes on mental health of residents are not fully understood.

From November to December 2019, we surveyed a Brazilian sample of physician residents on general aspects of residency programs as well as mental health issues [6]. During the first wave of COVID-19, in June 2020, we fielded a similar questionnaire to assess the impact of the pandemic on the training schedules, likewise mental distress. The objective of this study was to compare the COVID-19 cohort with the pre-pandemic group in terms of depression, anxiety, burnout and training schedules.

## Methods

This is a survey-based study with two cross-sectional independent convenience samples of physician residents from all geographical regions of Brazil and from medical, surgical and diagnostic areas of training. Both samples used online surveys which we posted on the page of the Associação Nacional de Médicos Residentes (Physician Residents National Association) on social networks (Instagram and Facebook); this webpage had around 10 thousand followers when we collected the data. For both cohorts, the participants needed to be physician residents at the time of data collection. The STROBE reporting guideline [9] was followed.

We collected the control sample in November and December 2019, and those results and methodological details were already published [6, 10, 11]. We applied it in this report to assess the impact of the first wave of COVID-19 on depression, anxiety and burnout of physician residents while controlling for confounders. All methodological description here is related to the COVID-19 cohort unless it is stated otherwise.

The sample was collected from 1 to 31st, June 2020, during Brazil’s first wave of COVID-19 cases. This survey was primarily aimed to assess the impact of the COVID-19 pandemic on the activities of residency training, availability of PPE and whether the residents were receiving additional remuneration for assisting COVID-19 patients.

### Survey questions

#### Residency training

The first section of the questionnaire asked about changes in residency training due to COVID-19 (displacement of residents from regular activities to COVID-19 areas, effect of reorganization on hands-on activities and classroom undertakings due to COVID-19), the perception of residents about COVID-19 impact on their training, and workload (weekly day off, duty hours in residency, and resting after overnight shifts). All those questions were close-ended.

#### Demographics

In the second section, we applied close-ended questions to collect demographic information that included: sex, age, postgraduate year (PGY), training specialty and geographical region of the residency program.

#### Mental distress

Depression and anxiety were assessed through the Patient Health Questionnaire-2 (PHQ-2) and the Generalized Anxiety Disorder-2 (GAD-2). Each of them comprised 2 Likert-type questions with options ranging from 0 to 3 (maximum score of 6 on each scale). Individuals who scored 3 or more in a specific domain were considered as positive screening—this approach has a sensitivity and specificity of 89% and 76% for depression [12], and 80% and 81% for anxiety [13].

Burnout was assessed by the two items version of the Maslach Burnout Inventory (MBI). It has two Likert-type questions (one for emotional exhaustion and another for depersonalization domain) with five options ranging from “no, I have not” to “yes, every day”. Participants who scored “yes, every day” or “yes, some days a week” in the specific domain were considered as positive screening in that domain—this approach covers an area under the receiver operating curve of 94% for emotional exhaustion and 95% for depersonalization, in relation to the original MBI domain [14]. Individuals who were positive in both domains were classified as having burnout. The tools and definitions of mental distress were the same in both cohorts.

### Ethical aspects

The COVID-19 cohort survey was launched a priori with no scientific reporting intention a priori, so the participants did not give their informed consent prior to fulfilling the survey. Ethical approval for the use of this dataset was obtained after data collection by the Maurício de Nassau University Center Ethics Committee (approval #4901115). These secondary data were anonymously given to this research team. The participants did not receive any financial support.

The pre-COVID-19 cohort (control) protocol was reviewed and approved by the Federal University of Pernambuco Review Board (approval #3314833). All volunteers gave their informed consent previous to enrolling in this study. Also, no benefits were given.

### Statistical analysis

We present data comparing the two cohorts. Quantitative variables are presented as median and interquartile range; Mann–Whitney test was applied. Qualitative variables are presented as absolute numbers and percentages; Fisher test was applied, and we provided the odds ratio (OR) and the respective 95% confidence interval (95% CI) for the main outcomes (depression, anxiety and burnout).

For controlling demographical and residency program-related differences between the cohorts, we applied a binary logistic regression model for each of the main outcomes. All demographical and residency program-related variables with *p* < 0.20 between the cohorts were entered into the regression model. We present the adjusted OR (aOR) and the respective 95% CI.

Sensitivity analysis was performed by bootstrapping 1 000 computer-generated samples for each multivariate model of the specific outcome. We reported the *β* coefficient (and the respective 95% CI) of the bias-corrected accelerated bootstrapping model.

All analyzes were performed in the IBM Statistical Package for the Social Sciences version 25 for macOS. An alpha coefficient of 5% (two-tailed) was used. There was no prespecified analysis plan for this study.

All answers were required to progress in the survey, hence there was no missing data.

## Results

There were 1 419 participants in the pre-COVID cohort and 524 residents in the pandemic group. Most of them were women (310/524, 59.2%), taking a surgical residency program (312/524, 59.5%), and from the South and Southeast region of Brazil (312/524, 59.5%). Table 1 depicts sample characteristics and compares both cohorts. The COVID-19 group was younger, but the median PGY was higher, and there were more participants in surgical programs.

**Table 1:** Sample characteristics

Variable	COVID-19 cohort (*n* = 524)	Control cohort (*n* = 1 419) [6]	*p*
Sex, female^a^	310 (59.2)	866 (61.0)	0.831
Age^b^	28 (26–30)	28 (27–30)	0.006
South-Southeast region^a^	363 (69.3)	972 (68.5)	0.783
PGY^b^	2 (2–3)	2 (1–3)	< 0.001
Specialty in training^a^			< 0.001
Surgical	312 (59.5)	610 (43.0)	
Medical	184 (35.1)	736 (51.9)	
Diagnostic	28 (5.3)	73 (5.2)	
Workload, up to 60 h/week^a^	431 (82.3)	717 (50.6)	< 0.001^c^
Weekly day off^a^	417 (79.6)	929 (65.5)	< 0.001^c^
Resting after overnight shifts^a^	401 (76.5)	1 024 (72.7)	0.092

^a^*n* (%)

^b^Median (interquartile range)

^c^Those differences persisted after controlling for confounders

COVID-19 cohort was collected in June 2020 (the first wave), and the control in November and December 2019

Furthermore, Table 1 also shows that duty hours up to 60 h per week of duty hours and having at least one day off per week (which are standards according to Brazilian laws) were more frequent in the COVID-19 cohort. These differences remained significant after controlling for demographics and residency program variables. The occurrence of resting after overnight shifts did not differ across the cohorts.

### Residency training re-organization to face COVID-19

The impacts of COVID-19 on residency programs organization are displayed in Table 2. Three-quarters of residents were reallocated from regular rotations to assist in COVID-19 areas; a decrease in duty hours of the original training program was reported by 90% of residents; classroom activities were adapted to virtual meetings or suspended for 95% of respondents. More than 3/4 of residents believed that they will need extra time for training after the COVID-19 re-organization, especially those from surgical and diagnostic training areas.

**Table 2:** Responses of COVID-19 cohort residents to survey questions

Variable	Grouping categories	Yes	%	*p*
Were residents displaced to COVID areas?		386	74.4	
	Surgical	220	71.4	0.110
	Medical	146	79.8	
	Diagnostic	20	71.4	
Do you believe that you will need an extra period of training after the end of pandemic due to COVID reorganization?		399	76.9	
	Surgical	247	80.2	0.038
	Medical	129	70.5	
	Diagnostic	23	82.1	
Have duty hours of the original residency training activities diminished due to COVID reorganization?				
	Yes, substantially	137	26.2	–
	Yes, partially	327	62.4	
	No	55	10.5	
Were there changes in classroom activities of your residency program due to COVID reorganization?				
	Yes, online	405	78.0	–
	Yes, canceled	85	16.4	
	No	29	5.6	

–: not applied

### Psychological distress

Table 3 summarizes data concerning psychological distress.

**Table 3:** Analysis of COVID-19 and pre-COVID-19 (control) cohorts regarding depression, anxiety and burnout in physician residents

Variable	COVID-19 (*n* = 524)	Control (*n* = 1 419) [6]	cOR (95% CI)	*p*	aOR (95% CI)^a^	*p*
Depression	282 (53.8)	666 (46.0)	1.32 (1.08–1.61)	0.008	1.64 (1.32–2.05)	< 0.001
Anxiety	296 (56.5)	803 (56.6)	1.00 (.81–1.22)	0.999	1.24 (0.99–1.55)	0.058
Burnout emotional	334 (63.7)	995 (70.1)	0.75 (0.61–0.93)	0.008	1.07 (0.84–1.35)	0.583
Burnout depersonalization	142 (27.1)	534 (37.6)	0.62 (0.49–0.77)	< 0.001	0.79 (0.62–1.01)	0.060
Burnout	137 (26.1)	525 (37.0)	0.60 (0.48–0.75)	< 0.001	0.77 (0.60–0.99)	0.039

c and aOR: crude and adjusted odds ratio

COVID-19 cohort was collected in June 2020 (the first wave), and the control in November and December 2019

^a^Controlled for age, PGY, specialty in training, residency duty hours, weekly day off and resting after overnight shifts

Positive screening for depression was found in 282/524 (53.8%) of residents. It represents an increase of 64% compared with the control cohort, after adjustment for confounders (age, PGY, specialty in training, residency duty hours, a weekly day off and resting after overnight shifts). Sensitivity analysis did not change those findings (bootstrapping *β* coefficient of 0.491, 95% CI = 0.268 to 0.724).

Anxiety was detected in 296/524 (56.5%) of our COVID-19 sample, which is the same as the pre-COVID-19 screening. Adjustment for confounders did not influence it. Bootstrapping *β* coefficient was 0.215 (95% CI = − 0.005 to 0.451).

Emotional and depersonalization domains of burnout were less frequent in the COVID-19 group than in controls in bivariate analysis, but the difference did not persist in multivariate models. However, overall burnout presented an adjusted difference of −23%  from COVID-19 to the control cohort, which was significant in the logistic regression. Sensitivity analysis corroborated this outcome (bootstrapping *β* coefficient of 0.259, 95% CI = 0.062 to 0.475).

## Discussion

In this study, we found that depressive symptoms were more frequent in physician residents during the first wave of the COVID-19 pandemic than before it; on the other hand, the frequency of burnout decreased, and anxiety rates did not change. It is interesting to observe that the organizational changes made to face the COVID-19 pandemic led to an overall duty hours reduction in residency training and having a day off per week became more frequent, among other modifications in residency programs. These changes were associated with residents’ self-reported need for additional training time. Our study provides some insights into environmental factors modulating mental distress.

Longitudinal studies with healthcare workers during this pandemic have shown enduring rates of mental distress [4, 15–17], although others found a reduction in these frequencies [18]. We highlight that the consequences related to psychological distress are vast, including increased rates of absenteeism [19], alcohol misuse, and medical errors [20], likewise may be related to an unsatisfactory learning experience [6], which is a keystone in residency training.

Positive screening for depression increased significantly between our surveys. It probably was influenced by a myriad of factors [3, 8], such as continuous exposure to the virus, limited PPE during the first wave, fear of being infected and transmitting it to family members, having personal relationships impaired, and death of family members and colleagues, among others.

Anxiety is one of the most frequent mental disorders among healthcare workers [1], especially those on the COVID-19 frontline [21]. However, anxiety symptoms did not change over time in our sample. It is already known that long working hours and poor supervision [6] are related to anxiety. Likewise, concerns such as limited information about how to manage those patients and the absence of specific treatment at that time point—mainly during the first wave of COVID-19—could lead to an anxiety state. Therefore, we believe that these factors might balance each other. To the best of our knowledge, other studies comparing pre- and pandemic periods on anxiety of residents or healthcare workers are lacking.

Burnout rates did not change from pre- to pandemic measures in cardiology [21], emergency medicine [22] and hospital-based, surgical or medical [23] residents. Another study reported a decrease in residents’ burnout of 13% per month during this pandemic [24], but the main factor related to burnout in residents is directly caring for patients with COVID-19 [25]. The association of burnout with duty hours was already known [6], and although workload had grown for many healthcare professionals, it had not for the majority of residents [7], including those surveyed here (about 1/3 of residents had their workload reduced to up to 60 h/week, and more 14.1% had a day off per week, as shown in Table 1). We believe that the burnout decrease we found—mainly related to the depersonalization domain—was due to this workload reduction.

On educational grounds, residency training was disrupted by the pandemic. All training areas were impacted, but residents of surgical and diagnostic specialties were more affected by those changes [7]—as we depict in Table 2, in which residents from these areas felt that training time should be extended. Elective surgeries and diagnostic procedures were canceled and some hands-on abilities could not be trained. At the same time, other diseases became less frequent in the urgency department [26], and the number of patients managed by residents and the general clinical experience were reduced [27]. Furthermore, we found 95% of the residents had their classroom activities canceled or changed to online meetings and 90% reported a reduction in the original residency training workload.

In the direction of narrow mental distress, psychological and psychiatric assistance should be made available for residents, as well as specific training to improve resilience and incentives towards a healthy lifestyle, including physical exercises [19]. Additionally, colleagues and staff should be aware of warning signs, such as avoidance behavior, unexplained absenteeism and alcohol abuse [20]. The training opportunities missed due to the redeployment of residents and faculty members should be addressed by policymakers and supervisors in order to guarantee the proper formation of specialists (profile and number of patients managed by residents, hands-on abilities, professional experience and soft skills). Additional training time for these residents should be discussed, in a case-by-case basis.

Our study has some limitations. The response and completion rates of the COVID-19 cohort could not be measured. Not all residents could be reached on the social media the survey was fielded, and a selection bias cannot be ruled out (according to the number of followers, we had 14.2% of respondents in the control group and 5.2% in the COVID-19 cohort), so generalizability might be limited. Although 3/4 of our samples were redeployed to COVID-19 areas, we did not measure the level of exposure to COVID-19 of each resident, which might influence mental health outcomes.

## Conclusion

Comparing the period before the pandemic and the during the first wave of COVID-19 in Brazil, depression frequency increased, anxiety did not change, and burnout decreased. Those variations are associated with training program re-organization to face COVID-19 and workload reduction, besides social changes.

The impact of the COVID-19 pandemic on training requirements should be specifically addressed by policymakers and supervisors in order to guarantee both the proper formation of specialists and the quality of life of residents.

## Data Availability

The datasets used and analyzed during the current study are available from the corresponding author on reasonable request.

## Abbreviations

PPE: Personal protective equipment
HCW: Healthcare workers
COVID-19: Corona virus disease 2019
